# Genetic Etiologies for Phenotypic Diversity in Sickle Cell Anemia

**DOI:** 10.1100/tsw.2009.10

**Published:** 2009-01-18

**Authors:** Martin H. Steinberg

**Affiliations:** Laboratory Medicine, Boston University School of Medicine, 72 E. Concord Street, Boston, MA 02118, USA

**Keywords:** fetal hemoglobin, alpha thalassemia, SNPs, genotype

## Abstract

The clinical course of patients with sickle cell anemia, a Mendelian trait, is characteristically highly variable. HbF concentration and the presence of a thalassemia are established modulators of the disease, but cannot account for all of its clinical heterogeneity. To find additional genetic modulators of disease, genotype-phenotype association studies, where single nucleotide polymorphisms (SNPs) in candidate genes are linked with a particular phenotype, have been informative. SNPs in several genes of the TGF-ß/MP superfamily, and some other genes linked to the endothelial function, and nitric oxide biology are associated with the subphenotypes of stroke, osteonecrosis, priapism, leg ulcers, pulmonary hypertension, and a more general measure of overall disease severity. Genome-wide association studies should help to confirm these observations and also to find hitherto unsuspected genetic modulators. Genetic association studies can have immediate prognostic value; they might also help to identify new pathophysiological pathways that could be susceptible to modulation.

## INTRODUCTION

Although a typical Mendelian single-gene disease, among patients with sickle cell anemia (homozygosity for *HBB* glu6val), there is substantial phenotypic heterogeneity. In this sense, the clinical expression of sickle cell anemia resembles a multigenic trait, such as diabetes or arteriosclerotic cardiovascular disease. In distinction to the typical multigenic disorder, the identical *HBB* mutation is present in each patient and is absolutely necessary to produce the disease. While necessary, the presence of the *HBB* gene product, sickle hemoglobin (HbS; $\alpha_2\beta^S{}_2$), alone is insufficient to account for the heterogeneity of the disease among patients. This suggests that variation in other genes, with environmental influences, are likely to modulate the phenotype of sickle cell anemia

Fetal hemoglobin (HbF) concentration, and the HbF distribution among erythrocytes, is the major genetic modulator of sickle cell anemia. The HbF level itself is genetically modulated. The coincidence of α thalassemia with sickle cell anemia is another powerful modulatory influence. Individually, other genetic modulators are likely to have smaller effects. However, as a group, genetic differences in many epistatic genes (and environmental factors) might have an important influence on morbidity and mortality.

## Genotypes of Sickle Cell Disease

Sickle cell anemia is not the sole genotype of sickle disease. Other common genotypes include HbSC disease (compound heterozygosity for the HbS and HbC genes), HbS-$\beta^+$ and $\beta^0$ thalassemia (compound heterozygosity for the HbS and different β thalassemia genes), HbSE disease (compound heterozygosity for the HbS and HbE genes), and HbSO Arab (compound heterozygosity for the HbS and HbO Arab genes). Other rare genotypes of sickle cell disease are also found. HbS concentration is a major pathophysiological factor in determining disease severity. Usually, but not always, compound heterozygous forms of sickle cell disease are clinically less severe than sickle cell anemia because of a lower concentration of HbS. Compound heterozygosity for the HbS and another β-globin gene mutation is an obvious cause of phenotypic heterogeneity and will not be discussed further. Nevertheless, each genotype of this disease has its own phenotypic diversity that is likely to be genetically modulated. Genetic association studies have almost uniformly been focused on sickle cell anemia.

## Genotype-Phenotype Association Studies

Three issues must be considered when evaluating genotype-phenotype association studies in sickle cell anemia. First, most genotype-phenotype association studies have not integrated the numerous clinical and laboratory dimensions of the disease into a single measure of disease severity. By modeling the likelihood of death with a network based on common clinical and laboratory findings, a risk of death within 5 years could be computed[1], When this likelihood was used as a severity score in a candidate gene-based genetic association study, genes involved in oxidative and vascular biology, fatty acid oxidation, and inflammation were associated with severity.

Second, one-SNP-at-a-time (SNP, single nucleotide polymorphism) and one-phenotype-at-a-time approaches to association studies are not likely to capture the complexity of genetic modulation. Bayesian networks are directed acyclic graphs in which nodes represent random variables and arcs define directed stochastic dependencies quantified by probability distributions. An analysis conditional on the phenotype reduces the complexity of the search, and can lead to discovery of larger sets of associations between SNPs and phenotype (for an introduction to these networks see http://en.wikipedia.org/wiki/Bayesian_network). For example, Bayesian networks were used to represent the mutual associations among many genetic variants to predict the likelihood of having a stroke in sickle cell anemia and showed that several candidate gene SNPs could predict the chance of stroke accurately[2].

Third, an impediment in genotype-phenotype association studies resides in the candidate gene-focused approach, where a gene is chosen *a priori* because of its potential pathophysiological importance. As a result, positive associations are gratifying, but must identify genes deemed interesting by the investigator; novel genetic modulators, beyond the imagination of the investigator, will not be found. In some studies, picking the "wrong" SNP or choosing insufficient numbers of SNPs in large genes might have led to false-negative results. Nonsynonymous coding region SNPs are an obvious first choice to examine, but are the least common. Recent studies suggest that by affecting protein folding, even synonymous SNPs might modulate a gene's function[3]. Genome-wide association studies (GWAS) have yielded surprising results in other diseases where the strongest associations of a genotype with a phenotype have occurred in gene-poor regions[4]. SNPs that effect gene expression might be even more important, but identifying all these by *in silico* analysis is difficult.

An unbiased assessment of genotype-phenotype relationships requires GWAS where hundreds of thousands to millions of SNPs are examined, freeing the investigator of the need to identify candidate modulators, and establishing any associations *a posteriori*. GWAS are just beginning in sickle cell anemia where patient numbers are limited compared with cardiovascular disease or diabetes, for example. Analytical methods are evolving and dealing with the problem of false-positive results that can be vexing when more than 1 million SNPs can be genotyped and case numbers are limited[5]. Other issues that must be considered in GWAS include linkage disequilibrium (LD) with causative polymorphisms, interpretation of associations with SNPs without an obvious function, gene-gene interaction, gene-environment interaction, false-negative results when the stringency for associations is set too high, and precise phenotype definition. Each of these elements of genotype-phenotype association studies is discussed in more detail in the following sections.

## FETAL HEMOGLOBIN

HbF ($\alpha_2\gamma_2$), whose γ-globin chains are encoded by *HBG2* and *HBG1*, is the best-known genetic modulator of sickle cell anemia. High concentrations of HbF dilute the amount of HbS, but importantly, by failing to be incorporated into the HbS polymer, HbF disrupts HbS polymerization. This attribute is not shared by HbA, HbC, HbE, or HbO Arab, which modify HbS polymerization solely by diluting the amount of HbS present. High concentrations of HbF, in general, reduce the severity of sickle cell anemia. HbF concentrations vary by an order of magnitude among patients with sickle cell anemia, suggesting that there is genetic regulation of γ-globin gene expression and HbF levels.

### Haplotypes of the β-Globin Gene–Like Cluster in Sickle Cell Anemia

The haplotype of the β-globin gene–like cluster is recognized by the pattern of SNPs in and surrounding these genes[6,7]. The $\beta^S$-globin gene is found on three major and one or two minor haplotypes, each originally localized to geographical regions of Africa, the Middle East, and the Indian subcontinent (reviewed in [8]) (Fig. 1A). A C-T polymorphism 158 base pairs (bp) 5' to *HBG2* (rs7482144) in carriers of Senegal and Arab-Indian haplotypes is strongly associated with HbF levels and with the concentration of $^G\gamma$-globin chains. Nevertheless, there is considerable diversity of HbF levels in carriers of this SNP, suggesting that additional modulatory elements are present; some of these are discussed below.

Lacking a reasonable hypothesis about how the haplotype of the β-globin gene cluster could modify disease severity, other than via an effect on HbF, it seems most reasonable to conclude that the effect of haplotype on the phenotype of disease is mediated through a cis-acting effect on HbF concentration. This, in turn, is associated with the presence of the 5' *HBG2* C-T SNP, and perhaps other cis-acting elements, as regions 5' to the γ-globin genes might have regulatory importance. The mechanism for this effect remains enigmatic.

Point mutations in the promoters of the γ-globin genes have also been associated with increased γ-globin gene expression, but these are rare and not a common cause of variation in HbF concentration[9]. Other polymorphisms that might be associated with HbF level have been called pre-γ framework[10].

#### *Effects of β-Globin Gene Cluster Haplotype on the Phenotype of Sickle Cell Disease*

Studies of small numbers of Africans with sickle cell anemia and different β-globin gene cluster haplotypes who had distinct hematological characteristics first suggested that haplotype could be a marker for the phenotypic heterogeneity of sickle cell anemia[11]. Environmental, nutritional, and infectious factors in many parts of Africa make it difficult to distinguish the role of haplotype in modulating the course of disease, but clinical differences have been noted. In population-based surveys, each haplotype was associated with clear hematological and clinical differences, although there is considerable heterogeneity within any haplotype. For example, as a rule, carriers of the HbS gene on the Senegal or Arab-Indian haplotype usually have the highest HbF level and packed cell volume (PCV), and the mildest clinical course. The Senegal and Arab-Indian haplotypes are the only ones with the C-T SNP 158 bp 5' to *HBG2* (originally known as the Xmn1 restriction enzyme site). Individuals with Bantu haplotypes have the lowest HbF level and PCV, and the most severe clinical course. Carriers of the Benin haplotype have intermediate features (reviewed in [12]) (Fig. 1B).

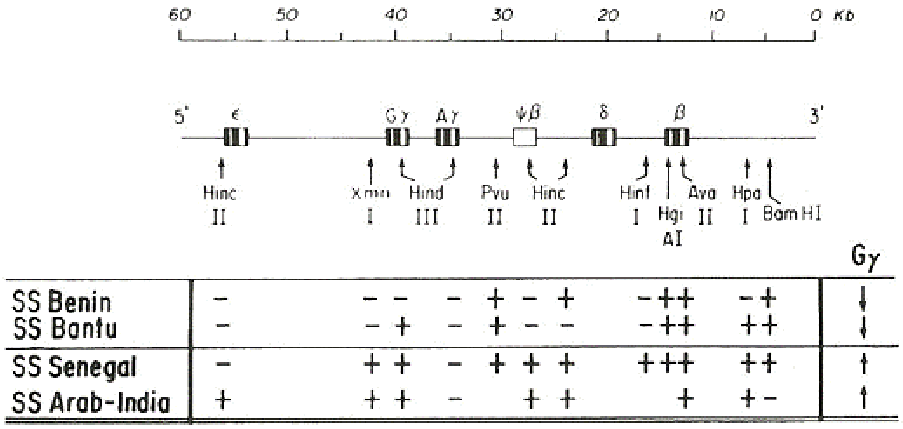

A

| | PCV | HbF | Clinical |
|---|---|---|---|
| **Senegal/Arab Indian** | highest | highest | best |
| **Benin** | intermediate | intermediate | Intermediate |
| **Bantu** | lowest | lowest | worst |

B

**FIGURE 1.** (A) Common haplotypes associated with the $\beta^S$-globin gene. Arrows indicate the approximate locations of the restriction endonuclease site that recognize the SNPs that characterize each haplotype, and the restriction enzymes originally used to define each site as being positive (cut by the enzyme) or negative (not cut). The Xmn1 site is the site of cleavage by this enzyme when a T replaces the wild-type C (rs7482144). (B) General clinical and laboratory findings in common haplotypes of sickle cell anemia. These are broad generalizations, derived from the data of many patients, and an individual might not be reflective of larger group.

Most of the detailed and larger studies of the clinical and hematological effects of haplotype in sickle cell anemia have been in regions where the HbS gene arrived by gene flow and, after many years of genetic admixture, patients in such regions are often compound heterozygotes for two different haplotypes. This, and other genetic admixture, complicates the interpretation of the association of haplotype with phenotype. Reports of the clinical and hematological effects of haplotype in sickle cell anemia should be viewed carefully, since often the patients studied were few in number, the patient's ages differed among series, clinical events might not have been sharply defined, and distinctions between haplotype homozygotes and heterozygotes were often not clearly delineated.

In longitudinal studies from the U.S., the Senegal haplotype was associated with fewer hospitalizations and painful episodes[13,14]. An effect of the Senegal haplotype on reducing episodes of acute chest syndrome was of marginal significance. Both gender and haplotype affect HbF levels in sickle cell anemia. Females with the Senegal haplotype and high HbF can have less hemolysis and therefore higher PCV[15]. In the Eastern Oases of Saudi Arabia, sickle cell anemia is associated with the Arab-Indian haplotype and high HbF. Most work suggests that the Arab-Indian haplotype is also associated with milder disease, although vasoocclusive events do occur[16,17,18,19,20].

The Bantu haplotype was associated with the highest incidence of organ damage and renal failure was strongly associated with this haplotype[21].

## GENETIC MODULATION OF HBF

HbF expression is regulated by complex interactions among genetic and epigenetic factors, providing many opportunities for modulation of HbF level in sickle cell anemia.

### Potential Regulatory Regions 5' to *HBB*

#### *Locus Control Region (LCR) and HbF*

The LCR has a major role in expression of the β-globin–like genes. Most studies of the influence of the LCR on HbF production have focused on 5' hypersensitive site (HS)-2, which is polymorphic among HbS-associated haplotypes. Nevertheless, SNPs in the LCR associated with HbF in sickle cell anemia have not been pinpointed[12].

Polymorphisms in a tandem repeat of the sequence $(TA)_xN_{10\text{-}12}(TA)_y$ that contains a Hox2 binding site were examined in 100 patients with sickle cell anemia aged 1–18 years[22]. Nearly 8% of chromosomes had a discordance in the HS-2 tandem repeat that was not characteristic of the cognate haplotype[23,24]. A region between –1445 and –1225 5' to the promoter of the *HBG2* gene was found to vary among haplotypes. Senegal-Benin chromosomes associated with modest HbF levels had a likely breakpoint for recombination upstream of –1500 bp 5' to the *HBG2* promoter. In contrast, when a high HbF was present with the Senegal-Benin chromosome recombination, the breakpoint was 3' to position –369 to –309. In sickle cell trait carriers with a Benin haplotype, when the normal chromosome had the HS-2 $(TA)_9 N_{12}(TA)_{10}$ structure, HbF (0.9%) and F cells (8.3%) were about twice as high as found with other configurations for this region[25]. These data suggest that HbF is influenced by elements 3' to HS-2 and 5' to the *HBG2* promoter.

F-cell numbers in Benin haplotype sickle cell trait carriers were more strongly associated with the $(TA)_9N_{12}(TA)_{10}$ configuration of HS-2 of $\beta^A$ chromosomes than the –158 C-T *HBG2* SNP[26]. Individuals with sickle cell trait do not have hemolytic anemia, so the results are less likely to reflect differential F-cell survival as found in sickle cell anemia, and more likely to estimate HbF production. While other cis-acting elements, for example, in additional phylogenetically conserved regions of the LCR outside the core sequences of its constituent hypersensitive sites, might partake in the regulation of γ-globin gene transcription, definitive associations have not been found[12].

The activity of constructs containing variant HS-2 enhancers derived from HbS chromosomes was studied to examine the functional effects of these polymorphisms[27]. In a multiplex assay permitting simultaneous analysis of three polymorphic cis-acting elements spanning 53 kb of the β-globin gene cluster, concordance between polymorphic alleles in γ- and β-globin gene promoters were identified. SNPs in HS-2 of the LCR were found juxtaposed to atypical cis alleles in the γ-globin gene promoter. Analysis of many such hybrid haplotype chromosomes suggested that polymorphisms in the γ-globin gene promoter exerted the dominant influence on HbF level in sickle cell disease[28].

#### *5' to the $^G\gamma$-Globin Gene*

A SNP, GA<u>T</u>A→GA<u>G</u>A, in a GATA site that is in a putative silencing element at nucleotide –567 5' of the $^G\gamma$-globin gene promoter was associated with increased HbF in two otherwise normal individuals. DNA protein binding assays showed that this GATA motif could bind GATA-1 transcription factor *in vitro* and *in vivo*. Truncation analyses of $^G\gamma$-globin gene promoter linked to a luciferase reporter gene revealed a negative regulatory activity present between nucleotides –675 and –526. In addition, the T-G mutation at the GATA motif increased the promoter's activity by two- to threefold in transiently transfected erythroid cell lines. The binding motif is uniquely conserved in simian primates with a fetal pattern of γ-globin gene expression. This GATA motif appears to have a functional role in silencing γ-

globin gene expression in adults. The T-G mutation in this motif disrupts GATA-1 binding and the associated repressor complex, abolishing its silencing effect and resulting in the up-regulation of γ-globin gene expression[29]. The functional importance of this site was supported by studies in transgenic adult β-YAC mice, where it was shown that during definitive erythropoiesis, γ-globin gene expression is silenced, in part, by binding a protein complex containing GATA-1, FOG-1, and Mi2 at the –566/–567 GATA sites of both γ-globin gene promoters. Chromatin immunoprecipitation assays showed that GATA-1, FOG-1, and Mi2 were recruited to the –566 or the –567 GATA sites of the γ-globin gene promoters when γ- expression was low, but not when these genes were being expressed[30].

Further, 5' to the $^{G}\gamma$-globin gene, between 1.65 and 1.15 kb, is a region of about 0.5 kb that contains four GATA-1 binding sites and Sp1 and CRE protein binding domains whose polymorphisms are associated with the β-globin gene cluster haplotypes[10]. This site was associated with differential binding of erythroid-specific and ubiquitous transcription factors. The strongest protein binding was associated with the Senegal pre-$^{G}\gamma$ framework, and the Benin haplotype–linked pre-$^{G}\gamma$ enhancer activity was sevenfold lower than the Bantu and Senegal type pre-$^{G}\gamma$ framework. The physiological significance of these findings remains unclear.

### Four Base Pair Deletion Linked to the $^{A}\gamma$ Gene

Only the Cameroon haplotype $\beta^{S}$ chromosome is associated with an AGCA deletion at nucleotides –222 to –225 5' to the β-globin gene that is always linked to the $^{A}\gamma^{T}$ allele. The effect of this deletion on γ-globin gene expression is controversial and unlikely to affect carriers of other haplotypes. In a study of sickle cell anemia, where a Cameroon haplotype was trans to a typical HbS haplotype, HbF levels, PCV, and mean corpuscular volume (MCV) were similar whatever the haplotype in trans[31]. It was also suggested that the 4-bp deletion was associated with decreased expression of not only the $^{A}\gamma^{T}$-globin gene, but also the $^{G}\gamma$-globin gene in cis.

### β-Globin Gene Silencer

Found –530 bp 5' to the β-globin gene is an AT-rich region with the core structure $(AT)_x(T)_y$, which is polymorphic and linked to the β-globin gene cluster haplotype. This element has been proposed as a β-globin gene silencer that might influence the expression of the β-globin gene by variably binding a putative repressor protein, BP1 (*DLX4)*, depending on the $(AT)_x(T)_y$ composition[32]. By modulating erythropoiesis, BP1 might also effect HbF production[33].

## Polymorphisms in Trans-Acting Regulatory Elements Regulating HbF Expression

### HbF-Related Quantitative Trait Locus (QTL)

Cis-acting regulation accounts for less than 25% of the variability of HbF in sickle cell anemia, suggesting the importance of trans-acting regulatory elements. The known putative trans-acting elements modulating HbF include four QTLs: the F-cell production locus, a QTL at Xp22 associated with F-cell number[34]; a QTL at 6q22.3-23.2 associated with F-cell numbers first described in an extended Asian-Indian family[35,36,37]; a putative QTL at chromosome 8q that appeared to interact with the –158 C-T SNP[38]; a QTL at 2p16.1, identified first in a GWAS of healthy adults mapped to *BCL11A*, a zinc-finger protein[39,40,41]. The 6q and 2p loci explain about half the variance in HbF in normal individuals and are also associated with HbF level in sickle cell anemia and β thalassemia. The 8q and Xp intervals were not associated with HbF in GWAS.

To refine the functional importance of the 6q QTL, direct sequencing of five protein-coding genes within a 1.5-Mb candidate interval of 6q23, *ALDH8A1, HBS1L, MYB, AHI1,* and *PDE7B* was done, but failed to detect mutations that could be associated with HbF modulation[42]. However, the expression profile of these genes in cultured erythroid cells of healthy adults with nongene deletion hereditary persistence of fetal hemoglobin (HPFH) found that two genes, *MYB* and *HBS1L*, were down-regulated. Transfection of K562 cells with cDNA of *MYB* and *HBS1L* showed that overexpression of only *MYB* inhibited γ-globin gene expression. Low levels of *MYB* were associated with low cell expansion and accelerated erythroid differentiation, suggesting that differences in the intrinsic levels of *MYB* might account for some variation in adult HbF levels by its effect on the cell cycle[43]. In Northern European families, polymorphisms within and 5' to *HBS1L* were strongly associated with F-cell levels, accounting for 17.6% of the F-cell variance. Although mRNA levels of *HBS1L* and *MYB* in erythroid precursors are positively correlated, only *HBS1L* expression correlated with high F cells, suggesting that *HBS1L* variants modulate HbF[44].

A QTL at chromosome 8q appeared to interact with the –158 C-T SNP to modulate HbF levels in the same Asian-Indian family where the 6q QTL was first discovered[36,38]. In more than 870 dizygotic twins, the effect of the 8q QTL on HbF was also conditional on the genotype of the –158 *HBG2* C-T SNP[45]. In candidate gene studies, SNPs in *TOX*, coding for a high-mobility group protein and located within the 8q QTL, were associated with HbF in both young and older patients (see below).

A candidate gene screening study in patients with sickle cell anemia showed associations of HbF with SNPs in *PDE7B, MAP7, MAP3K5*, and *PEX7* that abutted the 6q interval[46]. In further candidate gene association studies, panels of haplotype-tagging SNPs in the β-globin gene–like cluster, in QTLs on chromosomes 6q, 8q, and Xp, and putative HbF regulatory regions, were genotyped in two independent sickle cell anemia patient groups to study their association with baseline HbF levels. HbF concentration was modeled as a continuous variable using a novel Bayesian approach. In subjects aged 24 years or more, five SNPs in TOX (8q12.1), two SNPs in the β-globin gene–like cluster, two SNPs in the Xp QTL, and one SNP in chromosome 15q22 were associated with HbF. Four other SNPs in 15q22 were associated with HbF only in the larger dataset. Included in the 15q22-21 interval are *MAP2K1*, *SMAD3*, and *AQP9*. None of these genes has a known connection to HbF synthesis or erythropoiesis, this region is not a known QTL associated with HbF, and this work has yet to be confirmed independently.

When patients less than 24 years of age were examined, additional genes, including four with roles in nitric oxide (NO) metabolism, were associated with HbF level. By stratifying patients by age, these results also suggested that different genes might modulate the rate of decline of HbF and the final level of HbF levels in sickle cell anemia[47]. The results of this analysis confirmed prior work using more traditional analytic approaches[48].

The first results of GWAS seeking to link SNPs with HbF concentration have now been reported. SNPs in the QTL at 2p16.1 and mapped to *BCL11*were first found to be associated with F cells in healthy adults[39]. Similar associations of SNPs in *BCL11A* were found in Sardinian β thalassemia patients and by focused genotyping in more than 1,200 patients with sickle cell anemia[40].

With GWAS of 113 parents of Thai HbE-β thalassemia patients and 255 unrelated African Americans with sickle cell anemia, and by focused genotyping of 250 parents of β thalassemia major patients from Hong Kong, association of HbF and F cells were found with one of the same SNPs of *BCL11A* (Fig. 2). In the patients with sickle cell anemia, homozygotes for the C allele of SNP rs 766432 had an average of 7% HbF compared with 3% HbF in patients homozygous for the A allele[41]. These findings in at least four different populations are consistent with an aboriginal *BCL11A* variant in Yorubans that is highly conserved among ancestral populations, even as the haplotype blocks containing this gene have diverged.

## *SAR1A*

A small guanosine triphosphate (GTP)-binding protein, secretion-associated and RAS-related (*SAR1A*) protein is inducible by hydroxyurea and might play a pivotal role in induction of γ-globin gene expression

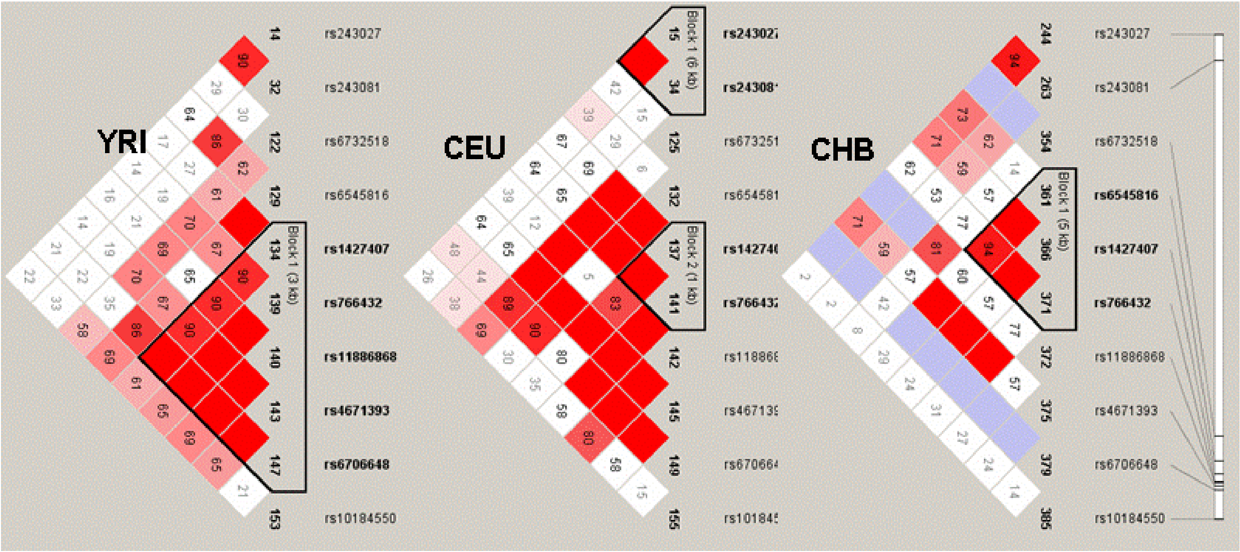

**FIGURE 2.** LD plots incorporating SNPs of *BCL11A*. Each square reports the value of D', with the standard LD color scheme: white D' < 1 and LOD < 2; blue D' = 1 and LOD < 2; shades of pink/red D' < 1 and LOD ≥ 2; bright red D' = 1 and LOD ≥ 2. YRI, trios from Ibadan, Nigeria; CEU, trios from Utah residents with northern and western European ancestry; CHB, Han Chinese from Beijing, China. Generated using HapMap data with the program HaploView 4.0. (From Sedgewick et al.[41])

via its role in erythroid maturation[49]. Polymorphisms in the *SAR1A* promoter were associated with differences in HbF levels or the HbF response to hydroxyurea in patients with sickle cell disease anemia[50]. Three previous SNPs in the upstream 5'UTR (–809 C-T, –502 G-T, and –385 C-A) were significantly associated with the HbF response in sickle cell anemia patients treated with hydroxyurea and four SNPs (rs2310991, –809 C-T, –385 C-A, and rs4282891) were significantly associated with the change in absolute HbF level after 2 years of treatment.

These genetic association studies have been augmented by functional analysis. *BCL11A* expression was inversely correlated with the expression of the HbF gene in K562 cells and human erythroid cell types representative of different developmental stages[51,52]. In K562 cells, *BCL11A* isoforms were expressed, while treatment of these cells with butyrate to induce *HBG* expression was associated with reduced *BCL11A* protein and mRNA. Increased expression of *BCL11A* caused more than a 50% reduction of *HBG* promoter transcription activity. *BCL11A* protein bound GGCCGG motif from nucleotides –56 to –51 of the *HBG* proximal promoter[51]. Its protein exists in complexes with the nucleosome remodeling and histone deacetylase (NuRD) corepressor complex, and the erythroid transcription factors GATA-1 and FOG-1. Abundant expression of full-length isoforms of *BCL11A* was developmentally restricted to adult erythroid cells. Transient or persistent knockdown of *BCL11A* caused robust induction of γ-globin gene expression[52].

*MYB* and *HBS1L* expression was simultaneously down-regulated in individuals with high HbF, while overexpression of *MYB* inhibited γ-globin expression. ChIP-chip data showed high levels of histone acetylation in the *MYB-HBS1L* intergenic region with a concentration in the *HMIP-2* block, indicating transcriptional activation in erythroid precursors. Potential cis-regulatory elements were identified in the same region as strong GATA-1 signals in coincidence with DNase I hypersensitive sites, suggesting a regulatory region in the *MYB-HBS1L* intergenic region that could control expression in the *MYB* locus[53].

## α THALASSEMIA AND HEMATOLOGICAL AND CLINICAL VARIABILITY

About a third of patients with sickle cell anemia have coincidental α thalassemia that is almost always a result of heterozygosity or homozygosity for the -$\alpha^{3.7}$ deletion[54]. These individuals have less hemolysis, higher PCV, lower MCV, and lower reticulocyte counts, and the changes are more pronounced in α thalassemia homozygotes than heterozygotes. By reducing the mean cellular HbS concentration and erythrocyte density, α thalassemia increases the sickle erythrocyte lifespan and, consequently, the PCV.

α Thalassemia decreased the risk of organ failure in sickle cell anemia patients with a Bantu haplotype. In Jamaicans, the absence of α thalassemia, coupled with a high HbF, presaged more benign disease[55]. The benefits and liabilities afforded by the presence or absence of α thalassemia in sickle cell anemia are very likely to be due to its effects on hemolysis. Some associations of α thalassemia and the clinical features of sickle cell anemia are shown in Table 1.

**TABLE 1**
**Effect of α Thalassemia on Some Clinical Complications of Sickle Cell Anemia**

| Phenotype | Effect of α Thalassemia |
|---|---|
| Survival | Probably little effect |
| Painful episodes | Permissive or little effect in sickle cell anemia |
| Stroke | Protective |
| Osteonecrosis | Permissive in sickle cell anemia |
| Acute chest syndrome | Unrelated or permissive |
| Heart | Cardioprotective |
| Cholelithiasis | Protective |
| Leg ulcer | Protective |
| Splenic function | Protective |
| Growth and development | Unrelated |
| Menarche | Unrelated |
| Priapism | Protective |
| Splenic sequestration | Permissive |
| HbF | Probably little effect |
| Albuminuria | Protective |

Note: The effects of α thalassemia are most likely a result of a reduction in sickle erythrocyte density and improved red cell survival. Heterogeneity among populations of patients with sickle cell anemia, in the ages of the patients studied and in the numbers of patients in each study, sometimes makes firm conclusions difficult. “Protective” denotes a reduction in the incidence or prevalence of a phenotype with α thalassemia. “Permissive” denotes an increased incidence or prevalence of a phenotype when α thalassemia is present. Modified from Steinberg[68].

Dysregulated NO homeostasis, a consequence of intravascular hemolysis, is likely to be responsible for some complications of sickle cell disease and other chronic forms of hemolytic anemia[56]. NO binds soluble guanylate cyclase that converts GTP to cGMP, relaxing vascular smooth muscle and causing vasodilatation. When plasma hemoglobin liberated from intravascularly hemolyzed sickle erythrocytes consumes NO, the normal balance of vasoconstriction:vasodilation is skewed toward vasoconstriction.

The subphenotypes of sickle cell anemia occur at different rates in different patients. Some complications, such as pulmonary hypertension, leg ulcers, priapism, and stroke, are associated with the intensity of intravascular hemolysis; others, such as acute painful episodes, acute chest syndrome, and osteonecrosis, are more closely related to sickle vasoocclusion and blood viscosity[57,58]. Concurrent α thalassemia was associated with a reduction in the incidence of elevated transcranial Doppler flow velocity, stroke, priapism, leg ulceration, and pulmonary hypertension, but had little effect on or increased the likelihood of developing osteonecrosis, acute chest syndrome, and acute painful episodes[57,59,60,61, 62,63,64,65,66].

Seventy-eight percent of sickle cell anemia patients were categorized as having either a "pain crisis" or "leg ulcer" phenotype. Neither α thalassemia nor the β-globin gene cluster haplotype appeared to influence the clinical events defining these phenotype groups[67].

## GENETIC POLYMORPHISMS AS PREDICTORS OF DISEASE SEVERITY

Neither HbF level nor α-globin genotype can fully explain the clinical and laboratory diversity of sickle cell anemia. Both HbF and α thalassemia impact the phenotype of disease by decreasing the polymerization tendency of HbS. HbF by virtue of exclusion from the polymer phase, and α thalassemia by reducing erythrocyte density and HbS concentration affect polymerization tendency. Polymorphic genes that could potentially affect the pathogenesis of this disease and modulate its phenotype were first selected because of their potential role in diverse pathophysiological events. These included pathways that mediate hemolysis, vascular remodeling, endothelial integrity, inflammation, oxidant injury, and NO biology.

Presently, most reported studies have examined only candidate genes and more comprehensive GWAS are just beginning[68]. To date, a unifying theme that is emerging from candidate gene studies is that polymorphisms in genes of the TGF-β/BMP pathway, a superfamily of genes modulating wound healing and angiogenesis along with many other functions, appear to be associated with several disease subphenotypes[69] (Fig. 3).

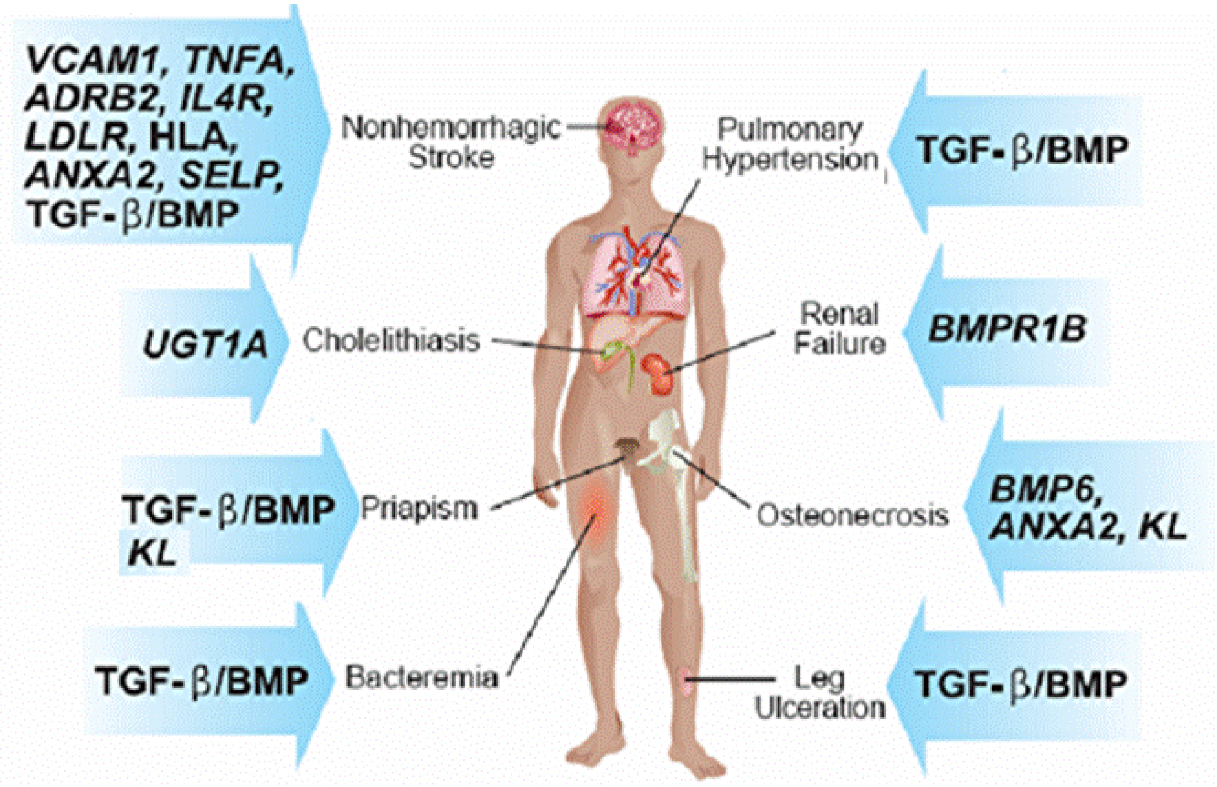

**FIGURE 3.** Analysis of SNPs in candidate genes suggest associations with subphenotypes of sickle cell anemia. For details of these associations and references, please see the text. (Modified from Steinberg[120].)

While an intriguing and an important beginning, the results of the data to be discussed need to be interpreted with several caveats. Many of the reported SNP association studies using various disease subphenotypes have examined relatively small numbers of patients, few studies have included independent patient groups for validation purposes, and with some exceptions, interactions among SNPs and the risk of a phenotype was not examined. Importantly, all of these studies are "discovery" science; they reveal genetic associations, but do not define causality. Other than speculation, these reports say nothing about the mechanism by which an associated polymorphic gene impacts the disease. This will be the next important step in defining genetic modifiers and turning their discovery into novel therapeutics.

## Painful Episodes

Acute painful episodes are the major clinical events of sickle cell disease[70]. The rate of painful episodes varies widely among patients; highest pain rates are found in patients with high PCV and low HbF. They occur less often in patients with hyperhemolysis, defined by the quartile of serum LDH level, and hyperhemolysis cases also have reduced survival[58]. Based on this observation, one might conclude that more severe hemolytic anemia with reduction of blood viscosity decreases the incidence of pain triggered by sickle vasoocclusion. Nevertheless, a possible paradox exists, in which painful events, associated with reduced survival[71], more commonly occur in patients with the lowest levels of hemolysis who have improved survival. Intriguingly, both observations were made using the same patient database[58]. This paradox is likely to be more apparent than real, and explained by different groupings of patients, varying patient ages in these groups, and dissimilar analytical approaches. Also, the dichotomization of sickle cell disease pathophysiology into vasoocclusive and hemolytic components[57] can only be used to espouse general principals, and as vasoocclusion and hemolysis are bound to be interrelated, they cannot be used dogmatically except for illustrative and didactic purposes.

A genetic basis for the heterogeneous distribution of painful episodes among patients has not been described. Case-control studies are problematic since nearly all patients will have pain, so that finding genes that modify the risk of pain alone using this approach will be difficult. A more innovative method of defining and analyzing a "pain phenotype" will have to be devised. Patient response to opioid analgesics varies considerably, and the efficacy of these drugs is known to depend on genetic variability of their catabolic enzymes and receptors[72]. How this might affect the treatment of the sickle cell acute painful episode is unknown.

## Stroke

Stroke has been the subject of the greatest number of genetic association studies. A familial predisposition to stroke in sickle cell anemia suggested that genetic modulation of this phenotype was possible[73]. Among the genes and SNPs associated with stroke in sickle cell anemia were two alleles of vascular adhesion molecule-1 (*VCAM1*): G1238C in the coding region of Ig domain 5 and T-1594C, an intronic SNP. The coding region SNP was protective, while the intronic SNP predisposed to small vessel stroke[74,75]. Preliminary studies also suggested that the *VCAM1* G1238C SNP was protective for developing high transcranial Doppler flow velocity, which is a strong predictor of stroke in children[76]. *VCAM1* is about 19,000 bp long, has nine exons, and at least 200 SNPs. Clearly, choosing the "right" SNP is not trivial and any association is likely to identify LD. Six SNPs in the intercellular adhesion molecule-1 and CD 36 genes (*ICAM1*; *CD36*) were not associated with stroke[74].

When stroke was subdivided into large and small vessel disease based on imaging studies, SNPs in the interleukin 4 receptor gene (*IL4R;* nonsynonymous coding region, S503P) predisposed to large vessel stroke, while tumor necrosis factor α gene (*TNFA;* noncoding G308A) and β adrenergic receptor 2 (*ADRB2;* nonsynonymous coding region, Q27E) SNPs were protective. In the small vessel stroke group, a low-density lipoprotein receptor (*LDLR;* untranslated region) SNP was protective. Homozygosity for the combination of *TNFA* –308 GG and the *IL4R* 503P heterozygosity was associated with predisposition to large vessel stroke[75]. HLA genes might be risk factors for vascular disease. In sickle cell anemia patients with cerebral infarction, different HLA alleles were associated with increased risk of and protection from stroke according to whether the stroke was a result of large or small vessel disease[77,78]. A continuum must exist in the endothelium from small to large blood vessels, making the pathophysiological basis of these observations puzzling.

Other genetic associations with stroke, such the C1565T mutant of the platelet glycoprotein IIIa (*ITPG3*) gene, angiotensinogen (*AGT*), cystathione B synthase *(CBS;* 278thr 68 bp insertion), cholesterol ester transfer protein (*CETP;* –628A), apolipoprotein C III (*APOC3;* –641A), the C677T polymorphism in 5,10-methylenetetrahydrofolate reductase (*MTHFR*), the plasminogen activator inhibitor-1 gene (*PAI1*), TGF-β receptor 3 (*TGFBR3),* and adenine cyclase 9 (*ADCY9*)[79,80,81,82], were all equivocal or negative; however, the sample size was usually insufficient for a definitive result.

To examine the interactions between genes and their SNPs, and to develop a prognostic model for stroke in sickle cell anemia, a Bayesian network was developed to analyze SNPs in candidate genes in 1,398 unrelated subjects with sickle cell anemia, 92 of whom had an overt nonhemorrhagic stroke[2]. SNPs in 11 genes and four clinical variables, including α thalassemia and HbF, interacted in a complex network of dependency to modulate the risk of stroke. This network of interactions included three genes (*BMP6, TGFBR2, TGFBR3*) in the TGF-β/BMP family and in SNPs in *SELP*. Using an independent validation set, the model predicted the occurrence of stroke in unrelated individuals with 98.2% accuracy, predicting the correct outcome for all stroke subjects and for 98% of the nonstroke subjects. This gave a 100% true-positive rate, a 98.14% true-negative rate, and a predictive accuracy of 98.2%.

Mechanistic studies are not a prerequisite for developing prognostic models and the predictive accuracy of this stroke model is a step toward the development of prognostic tests better able to identify patients at risk for stroke. The presence among the risk factors of genes already associated with stroke in the general population, such as *SELP* and genes of the TGF-β/BMP pathway, suggest that genetic factors predisposing to stroke in sickle cell anemia might also be operative in the general population.

Gene expression studies have also contributed to the understanding of the predisposition to stroke[83]. When subjects at risk for stroke, estimated by the presence of Circle of Willis disease or having had a stroke, were compared with controls, transcripts in genes of inflammation-related pathways expressed in blood-outgrowth endothelial cells were most strongly associated with stroke or predisposition to stroke.

## Priapism

SNPs in 44 candidate genes were examined for their association with priapism in 148 patients with sickle cell anemia with priapism and 529 patient controls that had not developed this complication. Polymorphisms in Klotho *(KL)* showed an association with priapism by genotypic and haplotype analyses[84]. *KL* directly or indirectly promotes endothelial NO production. A strong association was also found between the prevalence of priapism, the severity of hemolysis, and the presence of α thalassemia[63]. In another study of about 200 men aged more than 18 years (mean 32.4 years), 83 had a history of priapism. A candidate gene study in this smaller population failed to show an association of priapism with SNPs in *KL*; nevertheless, there were associations with SNPs in *TGFBR3, AQP1*, and the adhesion molecule *ITGAV*[85]. Further study of this population found that a single coding SNP in *F13A1*, the Factor XIII gene, was associated with priapism with an odds ratio of 2.43 for individuals with C/C compared with the C/G genotype[86].

## Osteonecrosis

Osteonecrosis, present in nearly half of all adults with sickle cell anemia, is one of the so-called vasoocclusive/viscosity-associated phenotypes whose prevalence might be increased by concurrent α thalassemia. The C677T polymorphism in *MTHFR* was found in 36% of adults with osteonecrosis, but only 13% of controls[87]. Nevertheless, other reports failed to confirm this association (reviewed in [68]). The C1565T SNP in platelet glycoprotein IIIa (*ITPB3*) and a polymorphism in *PAI1* were not associated with sickle osteonecrosis[88]. These studies all examined small numbers of patients and very few SNPs.

When 442 adult sickle cell anemia subjects with osteonecrosis were compared with 455 patient controls, individuals with osteonecrosis had a higher prevalence of coincident α thalassemia. Significant associations were observed with SNPs in seven genes (*BMP6, TGFBR2, TGFBR3, EDN1, ERG, KL, ECE1*). In follow-up studies, additional SNPs, equally distributed within the genes, were typed in these seven genes and a significant association with many SNPs in *KL* and *BMP6* was found[89]. Of the 18 SNPs typed in *KL*, 10 were significantly associated with osteonecrosis. Most of these SNPs were located in the 20-kb region representing the first half of the first *KL* intron and were in LD. Five of 14 SNPs in *BMP6* were associated with this phenotype. SNPs in *ANXA2* were genotyped because of an association of this gene with sickle cell stroke; six of 13 were associated with osteonecrosis. Most SNPs were in LD and were distributed throughout the intronic and 3' untranslated regions of the gene. The distribution of haplotypes of *KL, BMP6*, and *ANXA2* were significantly different between cases and controls.

Among its many functions that include roles in NO biology, *KL* is a glycosyl hydrolase that participates in a negative regulatory network of the vitamin D endocrine system. Bone morphogenetic proteins (BMP), including *BMP6*, are pleiotropic secreted proteins structurally related to TGF-β and activins, and are involved in bone formation and development. The actual mechanisms by which variants of any of these genes predispose to sickle cell–related complications are unknown.

## Leg Ulcers

Sickle cell leg ulcers are closely associated with the severity of hemolysis and, as a group, ulcer patients had lower PCV and higher levels of LDH, bilirubin, aspartate aminotransferase, and reticulocytes than did controls. The coexistence of α thalassemia reduced the likelihood of having leg ulcers. When 387 sickle cell anemia patients with leg ulcers were compared with 920 patient controls in a candidate gene association study, SNPs in *KL*, *TEK* (*TIE2*), a gene expressed in the endothelium and also associated with sickle cell stroke, and several genes in the TGF-β/BMP signaling pathway were associated with the presence of leg ulcers[64].

## Bacteremia

Infection, bacteremia, and sepsis are common events in sickle cell anemia, and in the non–sickle cell disease population, SNPs in candidate genes have been associated with an increased risk of sepsis in the general population. In a case-control study of sickle cell anemia, 145 anemia patients who developed bacteremia were compared with 1,248 control cases. SNPs and haplotypes of genes of the TGF-β/BMP pathway, such as *BMP6, TGFBR3, BMPR1A*, *SMAD6*, and *SMAD3,* were associated with bacteremia[90].

Other studies have suggested that the incidence of infection in sickle cell disease might be modulated by polymorphisms of the human leukocyte antigen system, the mannose-binding lectin receptor, the Fc receptor, and the haplotype of the β-globin–like gene cluster[91,92,93,94].

## Renal Disease

Patients with sickle cell anemia associated with Bantu haplotype chromosomes are more likely to develop renal failure and other vasoocclusive complications, perhaps because these individuals have the lowest levels of HbF[21,95].

SNPs in selected candidate genes are also associated with glomerular filtration rate (GFR). Estimated GFR was used as a phenotype of sickle nephropathy and modeled as a continuous trait. Tagging SNPs in about 70 genes of the TGF-β/BMP pathway were genotyped and four SNPs in *BMPR1B*, a BMP receptor gene, yielded statistically significant associations. Ten SNPs, *BMPR1B* SNPs, could be used to construct a haplotype. A region harboring three SNPs in strong LD yielded the most statistically significant association. The global statistic for this region reached a *p* value of 0.0087 and the haplotype AAG (prevalence 43%) was inversely associated with low GFR, while the GGA haplotype (41%) was significantly associated with high GFR. The TGF-β/BMP pathway was first associated with the development of diabetic nephropathy, which has some features in common with sickle cell nephropathy[95].

## Pulmonary Disease

### Pulmonary Hypertension

Pulmonary hypertension or pulmonary vascular disease has emerged as an important risk factor for premature death in patients with sickle cell anemia, but its genetic basis has only recently started to be studied[96]. Pulmonary hypertension is likely to be modulated by the effects of genes that control NO and oxidant radical metabolism, cell-cell interaction, vasculogenesis, and vasoreactivity. For example, mutations in BMP receptor 2 (*BMPR2*) and other genes have been associated with both familial and sporadic pulmonary hypertension[97,98,99]. In 111 symptomatic patients screened for pulmonary hypertension, an association of this subphenotype was identified for genes in the TGF-β/BMP superfamily, including *ACVRL1*, *BMPR2*, and *BMP6*[97]. Remarkably, this study of a patient group totally independent of patients previously reported found that *BMP6* SNP rs449853, previously associated with sickle cell stroke and bacteremia, was associated with pulmonary hypertension, as estimated by the tricuspid regurgitant jet velocity[2,90]. Independent validations of this type lend additional credibility to SNP association studies[100].

### Acute Chest Syndrome

Acute chest syndrome is a common vasoocclusive complication of sickle cell disease with important morbidity and mortality[101]. Its complex etiology, and the different predominant causes in children and adults, make this phenotype a difficult one to examine as it lacks the discrete nature of events, like osteonecrosis or leg ulcers. Both an increased and decrease risk of having acute chest syndrome was associated with a T786C SNP in the endothelial NO synthase gene (*NOS3*); one small study did not replicate these observations[102,103,104]. Exhaled NO levels were reduced in patients with acute chest syndrome compared with controls, and this was associated with the number of AAT repeats in intron 20 of *NOS1*[105]. In follow-up studies, the ATT repeat polymorphism in *NOS1* was associated with acute chest syndrome only in patients without asthma (itself, a risk factor for acute chest syndrome)[104]. In this study of 134 children, 64% had at least one acute chest syndrome and 36% had asthma. The patient number was small, the relationship between the number of *NOS1* ATT repeats and risk of acute chest syndrome was an unusual curvilinear one, the association was modest, at best, and a plausible reason why this polymorphism should be important only in acute chest syndrome patients without asthma was not obvious.

A T8002C SNP in the endothelin-1 gene (*EDN1*) was associated with an increased risk of acute chest syndrome in 173 children with sickle cell anemia detected at birth and followed longitudinally[103]. Small numbers of cases and controls were examined, only children were studied, and few SNPs were examined.

A candidate gene association study of acute chest syndrome was performed using data from 1,422 subjects with sickle cell anemia and has been reported in abstract form. Because the etiology and clinical course of acute chest syndrome in patients less than 4 years of age is different from that in older-aged subjects, the population was dichotomized into pediatric (defined as age ≤ 5 years) and older children and adults (aged > 5 years)[106]. There were 170 acute chest syndrome cases and 884 controls in patients aged less than 5 years and 388 cases and 819 controls in older individuals. Using time-to-first event in an age, gender, leukocyte, reticulocyte, and platelet count-adjusted analysis, and controlling for the false discovery rate, two SNPs were significantly associated with acute chest syndrome in both patient groups. These SNPs were in *TGFBR3* and in an unknown gene in LD with *SMAD7* that is also in the TGF-β pathway. Additional SNPs significantly associated with acute chest syndrome in younger cases were in *PIK3CG*, a member of the PI3/PI4-kinase family involved in cell-cell adhesion. Six additional SNPs were associated with acute chest syndrome in older cases, and were found in *SMAD1*, *KL*, *NRCAM*, and *SMAD3*. The *NOS1* ATT repeat polymorphism, *EDN1*, and *NOS3* SNPs discussed above were not examined in this study.

## Hyperbilirubinemia and Gallstones

Promoter polymorphisms in the uridine diphosphate-glucuronosyltransferase 1A (*UGT1A1*) gene are associated with unconjugated hyperbilirubinemia and Gilbert syndrome. Children with sickle cell disease had a higher mean bilirubin level if they carried the 7/7 *UGT1A1* genotype compared with the wild-type 6/6 or 6/7 genotypes; patients with the 7/7 genotype were more likely to have had a cholecystectomy. This suggested that symptomatic cholelithiasis is more common in carriers of this genotype[107,108]. Steady-state bilirubin levels are also influenced by the presence of α thalassemia and the HbF level[109,110]. The 7/7 and 7/8 genotypes were risk factors for symptomatic gallstones only in older subjects with sickle cell disease, and while coincident α thalassemia was associated with less hemolysis, it did not compensate for the *UGT1A1* promoter polymorphism[108,109]. α Thalassemia, no matter the *UGT1A1* genotype, was associated with reduced serum bilirubin levels[108,109,110,111,112].

## Erythrocyte Glucose-6-Phosphate Dehydrogenase (G-6-PD) Deficiency

G-6-PD deficiency is common in sickle cell anemia. Studies of the phenotype of combined G-6-PD deficiency and sickle cell anemia have given disparate results, but in a multi-institutional study, G-6-PD deficiency was not associated with differential survival, reduced hemoglobin levels, increased hemolysis, more pain crises, septic episodes, or a higher incidence of acute anemic episodes[113]. Using DNA-based methods to detect unequivocally the $GdA^-$ allele of *G6PD*, it was reported that the frequencies of $GdA^-$ and of the normal GdB and $GdA^+$ genes, were identical in patients with sickle cell anemia and controls[114]. Blood counts were similar in patients with and without G-6-PD deficiency, although the hemoglobin concentration was lower in sickle cell anemia with the $GdA^-$ gene. The prevalence of $GdA^-$ did not change with age.

Patients with sickle cell anemia have impaired flow-dependant and -independent vasodilation[115]. This might be a consequence of intravascular hemolysis, heme scavenging of NO with decreased NO bioavailability, and oxidant stress. Adequate availability of G-6-PD is needed to maintain both NO levels and preserve the proper redox milieu. It has been proposed that a G-6-PD–deficient phenotype could be present in critical vascular tissues in G-6-PD–deficient individuals and perhaps even in sickle cell disease patients with a normal G-6-PD genotype[116]. Relative G-6-PD deficiency might occur in the endothelium and play a role in the endothelium-related pathophysiology of disease. It has been hypothesized that the hyperaldosteronism of sickle cell anemia might impair vascular reactivity by decreasing endothelial G-6-PD activity[117]. Consistent with this, in more than 200 children with sickle cell anemia, G-6-PD activity was found to be an independent risk factor for transcranial Doppler flow velocity and cerebral vasculopathy[66].

## Compound Phenotypes and Integrated Measures of Severity

Few studies have successfully combined disease complications and laboratory variables and used this as a phenotype in genetic association studies. One small study of patients with histories of stroke, acute chest syndrome, osteonecrosis, and priapism showed that patients with complications had a significantly higher frequency of the platelet glycoprotein allele, HPA-5b, compared with controls[118]. An individual needed but a single complication to be included, most events were osteonecrosis, and only four individuals had more than a single phenotype.

To produce an integrated disease phenotype, a Bayesian network model was developed that described the complex associations of 25 clinical and laboratory variables, deriving a score to define disease severity as the risk of death within 5 years[1]. A candidate gene association study used this score as an integrative phenotype, studying the association of 798 SNPs in candidate genes in 741 sickle cell anemia patients, aged 18 years, split into mild and severe cases. Attention was focused on SNPs in those genes in which the set of selected SNPs had less than a 10% chance of random selection. This criterion selected 34 SNPs in eight genes. Included among these genes were *ECE1, KL, TOX, GSR, TGFBR3*, and *CSF2*[119]. Some of these observations were confirmed using the patients employed for validation in the original modeling of the severity score. Among the SNPs in genes associated with disease severity, some associations, like *TGFBR3*, confirmed previous findings in stroke and pulmonary hypertension in subjects with sickle cell anemia, and supported the speculation that dysregulation of the TGF-β/BMP signaling pathway might play a major role in the modulation of disease severity. Other genes where SNPs were associated with severity, like *ECE1* and *KL,* are expressed in the endothelium or modulate endothelial function. Some associated genes play a less obvious role in the pathobiology of disease, but were strongly associated with the phenotype of severity. SNPs in these genes were also associated with normal aging in a group of nearly 1,000 individuals with exceptional longevity compared with controls. Perhaps increased oxidative stress and the relentless progression of vasculopathy in sickle cell anemia cause accelerated tissue damage that might be modulated by genes that affect the normal aging process.

In GWAS reported in abstract, 119 sickle cell anemia patients with severe disease (severity score $\geq$ 0.57) were compared with 553 milder cases (score < 0.57)[1] using Bayesian tests of association. Forty-nine SNPs were associated with a Bayes factor > 1000. Among the associated regions were the HbF QTL at 6q23 and genes like *CENPF* and *CHDB*[121]. The former gene plays a role in cell cycle regulation, while the later is involved in endothelial cell migration and proliferation. Some of these associations were validated in a smaller independent patient group.

As the results of unbiased GWAS are added to capture polymorphisms not included in candidate gene studies, a predictive network with even greater reliability than one using only clinical and laboratory variables might be developed.

## CONCLUSIONS

Genetic association studies based on analysis of candidate genes have suggested genes and pathways that might be the focus of resequencing efforts and functional analysis in model systems to discover the mechanisms of genetic modulation. GWAS hold the promise of providing a more thorough appreciation of the genetic diversity that underlies phenotypical heterogeneity. Nevertheless, positive findings from this approach will need to be confirmed and mechanisms studied. The near-term results of genotype-phenotype studies will likely be the ability to provide better prognostication to reduce the uncertainty associated with therapeutic decisions, such as the use of long-term transfusion to prevent stroke or the employment of multiple agents to lessen the adverse outcome associated with pulmonary hypertension. Ideally, and currently not refined sufficiently to be feasible, this information could be had antenatally and a personalized lifelong care plan formulated. The later-term goal of genetic association studies is to identify genes and pathways that might be therapeutically manipulated in novel treatment approaches.

## ACKNOWLEDGMENT

Supported by NIH grants R01 HL68970, R01 HL87681, R01 DK069646, and HL 70735.
